# The Skin in Spring-Time

**Published:** 1907-04-27

**Authors:** 


### Dermatology.

### THE SKIN IN SPRING-TIME.

Just about this time of year the practitioner will be pretty sure to come across a certain number of cutaneous disorders which owe their origin more or less directly to the climatic and other conditions. Few of them, happily, are of really serious import, but, especially in children, their recognition and treatment may prove of some difficulty. Such eruptions may be grouped as follows: (*a*) catarrhal, (*b*) toxic, (*c*) vaso-motor.

The skin is no less susceptible to changes of external temperature than the throat or lungs, so that it is not at all surprising that it should be attacked with some inflammatory disorder as the result of injudiciously discarding some, or all, winter clothing before "May is out."

### Eczema as a Cutaneous Reaction.

There is a growing tendency to regard eczema, which is really a catarrhal dermatitis, as a reaction on the part of the skin to some irritating agent, whether this act from within or from without the body. The use of some wholly unsuitable soap, or the direct action of a cold current of air upon the face, may determine a "reaction" which is clinically recognised as an attack of eczema. The face most commonly suffers in this way, and if the inflammatory process be severe the case may easily pass for one of erysipelas, but there is not the characteristic advancing border nor the constitutional disturbance met with in the latter affection. In these cases there is an almost complete absence of papule-formation, the chief manifestation being an acute erythema. The primary indication in treatment is to soothe and protect the inflamed skin, and lotions of calamine, made without glycerine, or simple Goulard water, may be applied in the earliest stages. When exudation has commenced, a creamy emulsion of the oleates of zinc and bismuth, 20 grains of each, with 30 grains of the carbonate of magnesium, in an ounce of sweet almond oil, will be more acceptable. The oleo-palmitate of zinc, an impalpable powder, is another most useful application. All washing with soap must be prohibited, but gentle bathing of the affected parts with tepid milk and water, with a little fine oatmeal, may be allowed. The kidneys and bowels should be encouraged to act well at the same time.

Some people get severe irritation of the skin every spring, accompanied by an outbreak of small papules, sometimes hardly visible, upon the face, neck, and arms. This is another form of catarrhal dermatitis, or eczema, closely connected with the next, or toxic, group of cutaneous disorders. The condition is worse in the evening and also after meals. Constipation is generally present, and symptoms of dyspepsia may also assert themselves. A careful overhauling of the diet and attention to the bowels are essential, strong tea and any excess of sweet foods being absolutely forbidden. An ointment of ichthyol, 10 grains, with the same quantity of zinc oxide, in one ounce of vasogen or lanoline, may be advantageously applied at night.

### The Urticaria of Infants.

Common papular urticaria in young children is a most troublesome thing. The older dermatologists knew it under the name of "Lichen urticatus" (Willan and Bateman), and, indeed, many of the lesions are distinctly lichenoid in aspect. True lichen planus is exceedingly rare in infancy, so that special attention should be paid to the mode of onset of the papules. The mother will usually state that the spots "begin like heat-bumps"; they are very irritable and quickly become surmounted with a minute blood-crust, the result of rubbing and scratching. When the papules retrogress they assume a flattened and even shiny character, but other lesions as wheals and definite papules will be sure to be seen. The improper feeding of infants, especially with patent foods containing an excess of starch, or with bread-jelly and sponge cakes, at an age when their digestive ferments cannot possibly cope with amylaceous matter, is largely responsible for the appearance of the eruption, but it is also a fact that cases are brought up to our skin clinics in greatest abundance in the spring and early summer. Enlargement of the inguinal and cervical glands does not occur in this affection, but this symptom is commonly present in the milder varieties of prurigo, with which papular urticaria may be easily confounded. Weak tar lotions—*e.g.*, cyllin, 1 in 2,000, or the liquor carbonis detergens, half a drachm to a pint of warm water, are useful to allay irritation, after which an ointment containing a little ichthyol (5 grains) or beta-naphthol (8 grains) to one ounce of vaseline may be applied. The effect of a nightly powder of half a grain of hyd. cum cretâ with a little sodium bicarbonate is often magical.

Many cases of acne are combined with true urticaria, linking, as it were, the toxic with the vaso-motor disorders. There can be no hard and fast rule for the treatment of these borderland cases, as the underlying cause in each must be diligently sought for. If saline purgatives and stomachic remedies do not improve matters, recourse may be had to the vaso-motor tonics, such as minute doses of ergot, or to calcium lactate, where there is deficient power of blood-coagulation.

A fourth class of cutaneous affections may be described as those which tend to recur, with more or less constancy, every spring, such as psoriasis, and a few special eruptions of a bullous type. With regard to the former complaint it has been found by Abraham that nearly 50 per cent. of the cases presented themselves at skin clinics for the first time during the months of April to June inclusive. The so-called *Prurigo hiemalis* has been shown by Pye-Smith to be specially frequent during the cold east winds of spring.

